# Ultrafast Random Number Generation Using Broadband Polarization Chaos in QD Spin-VCSELs

**DOI:** 10.3390/s26092588

**Published:** 2026-04-22

**Authors:** Christos Tselios, Panagiotis Georgiou, Christina (Tanya) Politi, Dimitris Alexandropoulos

**Affiliations:** 1 Department of Electrical and Computer Engineering, University of Peloponnese, 22100 Patras, Greece; 2 Department of Materials Science, University of Patras, 26504 Rion, Greece

**Keywords:** polarization chaos, polarization dynamics, spin lasers, quantum-dot spin-VCSELs

## Abstract

Semiconductor lasers have been widely employed in chaos-based information processing due to their ability to generate enhanced chaotic bandwidths. In this study, we investigate broadband polarization chaos in optically injected QD spin-VCSELs and their ability to act as high-speed physical entropy sources for random number generation (RNG). We achieve chaotic bandwidths approaching 50 GHz per polarization mode using elliptical injection. With optimized conditions and post-processing, we demonstrate RNG at rates of up to 240 Gb/s. The quality of the generated random sequences is evaluated using multiple statistical metrics, including entropy estimation based on the NIST SP800-90B framework, uniqueness analysis using Hamming distance, and bias assessment through autocorrelation and histogram analysis. In addition, the influence of different polarization injection schemes on randomness is examined using the NIST SP800-22 statistical test suite. These results highlight the potential of QD spin-VCSELs as compact and ultrafast sources for RNG in secure communication systems.

## 1. Introduction

Ultrafast and scalable RNG is crucial for modern cryptography, secure communications, and information security. Traditionally, RNG methods rely on sources such as amplified spontaneous emission (ASE) from Erbium-doped fiber amplifiers (EDFAs) [1], quantum mechanisms [2], random telegraph noise [3], diffusive memristors [4], and spintronics [5] to ensure randomness. However, these approaches often suffer from high complexity, specialized components, and limited scalability, increasing cost while restricting speed. To address these limitations, optical chaos [6] has been proposed as a promising alternative. Chaotic lasers can exceed electronic bandwidth constraints, enabling ultrafast RNG. Although chaos is deterministic in nature, the underlying nonlinear laser dynamics, combined with the quantum noise of spontaneous emission, can lead to effectively non-deterministic behavior while maintaining very high generation speeds.

Several approaches have been explored to push RNG rates into the terabit per second (Tb/s) regime by coupling multiple lasers [7,8]. However, these methods introduce significant complexity and scalability challenges, motivating alternative physical processes with inherently faster dynamics. A promising direction is parallel RNG, which exploits spatiotemporal dynamics in specially designed cavities within a single laser diode [9], chaotic micro-ring resonators [10], broad-area semiconductor lasers [11], and self-chaotic microlasers [12]. Another important factor related to speed is minimizing electronic bottlenecks in practical implementations, motivating the development of all-optical RNG schemes [13,14].

The concept of parallel RNG aligns with the inherent dual linear polarization modes in vertical-cavity surface-emitting lasers (VCSELs), offering an opportunity for ultrafast RNG through multichannel chaotic signal expansion [15,16]. These works demonstrated how the chaotic components can be efficiently utilized through XOR-based post-processing to achieve high-speed and statistically robust RNG. However, further investigation is required to refine post-processing techniques that fully exploit both polarization channels for parallel RNG. Zhang et al. [17] leveraged a 50 GHz chaotic bandwidth and four-LSB extraction using a 160 GS/s digital oscilloscope to achieve 640 Gb/s.

VCSELs leveraging polarization dynamics have been employed in diverse applications, including polarization-encoded Ising machines [18], photonic time-delay reservoir computing [19], and chaos generation, where the light’s polarization state exhibits deterministic chaotic dynamics [20]. More recently, polarization chaos in VCSELs has also been explored in optical sensing architectures, including polarization-multiplexed detection schemes for LiDAR [21]. However, various techniques, such as optical injection [22] and mutual coupling [23], have been employed to enhance the complexity and bandwidth of polarization chaos, achieving broadband polarization chaos up to 40 GHz. More recently, intensity-modulated optical injection [24] has further extended the chaotic bandwidth to 40–50 GHz [25,26]. These approaches have demonstrated strong potential for high-speed RNG, crucial for encryption and secure communications. Another widely used approach for generating complex laser chaos is optical feedback combined with optical injection [15,27]. However, this method suffers from time-delay signatures (TDSs), which compromise security in chaos-based communication systems [28] and increase system complexity.

The injection of spin-polarized carriers in spin-VCSELs introduces spin-dependent carrier coupling, either through electrical injection using magnetic contacts or through optical pumping with polarized light. This results in faster polarization dynamics, making spin-VCSELs highly suitable for optical communications [29,30] and photon-enabled computing applications [31,32,33]. The aforementioned coupling, together with nonlinear carrier–photon interactions, the inherent polarization mode competition induced by cavity birefringence, and the external perturbations such as optical injection, govern the emergence of complex nonlinear dynamics. Spin-VCSELs therefore provide a compelling alternative for chaos-based applications in terms of security and chaotic bandwidth.

Recent advances in spin-VCSEL technology have demonstrated their ability to generate broadband polarization chaos, further expanding their potential for high-speed applications. Two key approaches, i.e., spin-VCSELs with optical feedback and optical heterodyning of two free-running spin-VCSELs, were shown to achieve chaotic bandwidths exceeding 30 GHz [34]. Additionally, a master–slave spin-VCSEL injection scheme was demonstrated to generate a single chaotic signal with bandwidths reaching up to 50 GHz [35]. These developments have facilitated several real-world applications, including 4 Gb/s chaos-based encrypted transmission [36], 1.34 Gb/s physical key distribution via chaos synchronization [37], and chaos-based decision-making, where the multi-armed bandit problem was solved using a dual-state QD spin-VCSEL [38]. Our research group proposed a 240 Gb/s RNG scheme based on optically injected QD spin-VCSEL [39].

This study extends our previous work on optically injected QD spin-VCSELs for RNG by introducing a nonlinear dynamical characterization of the device, along with a systematic performance evaluation as a physical entropy source. In particular, we present a birefringence rate optimization analysis aimed at enhancing the chaotic bandwidth, and undertake a performance comparison of orthogonal, parallel, and elliptical optical injection schemes in terms of chaotic bandwidth, statistical pass rate, and certified entropy. Our method demonstrates high-speed chaotic bandwidths, achieving polarization chaos bandwidths of up to 48 GHz per polarization component under optimized device conditions. To establish QD spin-VCSELs as a secure and ultrafast RNG source, we conduct chaotic bandwidth simulations and evaluate randomness using certified entropy analysis following the NIST SP800-90B statistical tests [40]. Additionally, we apply widely used methods for randomness validation such as statistical bias analysis, autocorrelation functions, and Hamming distance evaluations. Furthermore, we conduct NIST SP800-22 test suite evaluations on 1 Gb sequences to rigorously assess randomness quality [41]. Our results demonstrate that QD spin-VCSELs can achieve RNG rates of up to 240 Gb/s, with potential for even higher rates through optimized post-processing techniques. These findings further highlight the potential of spin-VCSEL-based RNG approaches for realizing compact, integrable physical entropy sources suitable for secure distributed sensor networks and photonic IoT platforms.

## 2. Materials and Methods

### 2.1. Theoretical Model

Our theoretical analysis is based on the spin-flip model (SFM) coupled rate equations, which were shown to be in close agreement with experimental results for both short-wavelength [29] and telecom VCSELs [30,42]. In this work, the model is applied to optically injected QD spin-VCSELs [39] and is extended to include spontaneous emission noise. The inclusion of this term is essential, as in its absence the simulated chaotic dynamics remain fully deterministic and cannot accurately capture the intrinsic unpredictability required for a physical entropy source. The rate equations read:

(1) $$\begin{array}{c} \frac{dE_{x}}{dt}=\kappa \left(1+i\alpha \right)\left[\frac{1}{2}\left(n_{QD}^{+}+n_{QD}^{-}\right)E_{x}+\frac{1}{2}\left(n_{QD}^{+}-n_{QD}^{-}\right)iE_{y}-E_{x}\right]-\left(\gamma_{a}+i\gamma_{p}\right)E_{x}+k_{inj}E_{inj,x}e^{i\omega_{inj}t+\delta }+F_{x} \end{array}$$

(2) $$\begin{array}{c} \frac{dE_{y}}{dt}=-i\kappa \left(1+i\alpha \right)\left[\frac{1}{2}\left(n_{QD}^{+}-n_{QD}^{-}\right)E_{x}+\frac{1}{2}\left(n_{QD}^{+}+n_{QD}^{-}\right)iE_{y}-iE_{y}\right]+\left(\gamma_{a}+i\gamma_{p}\right)E_{y}+k_{inj}E_{inj,y}e^{i\omega_{inj}t}+F_{y} \end{array}$$

(3) $$\begin{array}{c} \frac{dn_{WL}^{\pm }}{dt}=\eta^{\pm }\gamma_{n}+h\frac{\gamma_{n}}{2}-\gamma_{0}n_{WL}^{\pm }\left(\frac{h-n_{QD}^{\pm }}{2h}\right)\mp \gamma_{j}\left(n_{WL}^{+}-n_{WL}^{-}\right) \end{array}$$

(4) $$\begin{array}{c} \frac{dn_{QD}^{\pm }}{dt}=\gamma_{0}\frac{n_{WL}^{\pm }}{h}\left(h-n_{QD}^{\pm }\right)-\gamma_{n}\left(h+n_{QD}^{\pm }\right)\mp \gamma_{j}\left(n_{QD}^{+}-n_{QD}^{-}\right)-2\gamma_{n}n_{QD}^{\pm }\left(\right|E_{x}{|}^{2}+|E_{y}{|}^{2}\mp iE_{x}{E_{y}}^{*}\pm i{E_{x}}^{*}E_{y}), \end{array}$$

where the linearly polarized complex field in the *x* and *y* directions are $E_{x}$ and $E_{y}$, respectively. In these equations, $E_{inj,x}=E_{inj}sin\left(\theta_{p}\right)$ and $E_{inj,y}=E_{inj}cos\left(\theta_{p}\right)$ are the amplitudes of the injected light in *x* and *y* polarization, respectively, while $k_{inj}$ is the injection rate and $\omega_{inj}$ stands for the angular frequency detuning. The injection level $P_{\mathrm{inj}}$ is defined as the injected optical power normalized to the output power of the solitary VCSEL, expressed in decibels (dB) [42]:

$$P_{\mathrm{inj}}=10\log\left(\frac{|E_{\mathrm{inj}}{|}^{2}}{|E_{\mathrm{sol}}{|}^{2}}\right),$$

$E_{\mathrm{sol}}$ is given by:

(5) $$|E_{\mathrm{sol}}{|}^{2}=\frac{\eta }{1-\frac{\gamma_{a}}{\kappa }}-1.$$

While absolute optical powers cannot be directly determined from the normalized model, the range of injection levels considered in this work (e.g., −26 dB to 10 dB) corresponds to regimes commonly explored experimentally in spin-VCSELs under optical injection, including both weak and strong injection conditions. The state of polarization of the optical injection is described by the auxiliary angle $\theta_{p}$ and the phase term δ as found in [43]. The remaining SFM parameters for the QD spin-VCSEL are defined as follows: κ is the photon decay rate, α is the linewidth enhancement factor, $\gamma_{n}$ is the carrier recombination rate, $\gamma_{0}$ is the capture rate from the wetting layer (WL) into the quantum dots (QDs), $\gamma_{j}$ describes the spin relaxation rates for both the WL and QDs, $\gamma_{p}$ is the birefringence rate, $\gamma_{a}$ is the dichroism rate, and *h* is the normalized gain coefficient, which can be expressed as $h=v_{g}\Gamma aN_{Q}D\tau_{p}$, where $v_{g}$ is the group velocity, Γ is the optical confinement factor, *a* is the differential gain, $N_{\mathrm{QD}}$ is the quantum dot density per unit volume, and $\tau_{p}={\left(2\kappa \right)}^{-1}$ is the photon lifetime. Within the normalized SFM framework, these parameters should be interpreted with care, as they provide an effective description of the interplay between material-dependent properties and cavity dynamics without explicitly resolving the underlying device structure. To account for spontaneous emission in the VCSEL, the final term in Equations (1) and (2) is expressed as

$$F_{x,y}=\sqrt{\beta_{\mathrm{sp}}\;\gamma_{n}\;n_{\mathrm{QD}}^{\pm }}\;\xi_{x,y}\left(t\right),$$

where $\beta_{\mathrm{sp}}$ represents the spontaneous emission factor. The fluctuations of this emission are described by the complex Gaussian noise processes $\xi_{x}\left(t\right)$ and $\xi_{y}\left(t\right)$, which have zero mean and unit variance. Their statistical properties follow

$$\langle \xi_{i}\left(t\right)\;\xi_{j}^{*}\left(t^{\prime }\right)\rangle =\delta_{ij}\;\delta \left(t-t^{\prime }\right),$$

ensuring that the noise components are uncorrelated in both time and polarization.

The pump parameters are the normalized pump intensity η, which is equal to 1 at threshold, and the pump ellipticity *P*. The total pump intensity is defined by $\eta =\eta_{+}+\eta_{-}$, where $\eta_{+}$ and $\eta_{-}$ are RCP and LCP normalized pump components, and the pump polarization ellipticity is defined by:

(6) $$P=\frac{\eta_{+}-\eta_{-}}{\eta_{+}+\eta_{-}}$$

### 2.2. Tools for the Visualization and Analysis of the Dynamics of Spin-VCSELs

To comprehensively analyze the nonlinear dynamics and randomness properties of optically injected QD spin-VCSELs, we employ a combination of bifurcation analysis, Largest Lyapunov Exponent (LLE), chaotic bandwidth mapping, and statistical randomness tests. These methods allow us to characterize stability transitions, quantify chaotic behavior, and validate the quality of extracted random bit sequences.

Bifurcations are qualitative changes in a system’s dynamics that provide insights into equilibria, periodicity, or chaos. A 1-D bifurcation diagram tracks changes in a system’s behavior as a single control parameter varies, typically displaying parameter values on the x-axis and intensity extrema on the y-axis.

The LLE is a widely used tool for investigating the dynamics of spin-VCSELs, and it has been extensively applied in previous studies of semiconductor lasers [44]. The LLE measures how quickly initially nearby trajectories in a chaotic system diverge from each other over time. It plays a crucial role in defining stability boundaries and quantifying chaotic behavior. A negative LLE indicates a stable point or limit cycle, and a value near zero represents periodic oscillations, while larger positive values signify complex dynamics, often leading to chaos. The LLE in this work was computed by integrating Equations (1)–(4) in Matlab (R2024b) using the ode45 solver (explicit Runge–Kutta (4,5) method with adaptive step-size control). The maximum time step was limited to Δt=1ps. The total simulation time was 300ns, with the first 30ns discarded as transient.

Heatmaps are employed to illustrate the sensitivity of QD spin-VCSELs to variation of key parameters. Meanwhile, the impacts of frequency detuning $\left(\Delta_{f}=\omega_{inj}/2\pi \right)$ and optical injection level $\left(P_{inj}\right)$ on chaotic bandwidth enhancement are investigated through contour maps, where the color bar represents the chaotic bandwidth. The latter is calculated from the Power Spectral Density (PSD) using the Fast Fourier Transform (FFT) of the time series solutions of Equations (1)–(4). Here, the bandwidth is defined as the range between DC and the frequency that contains 80% of the spectral power [22].

In addition to analyzing the chaotic bandwidth of spin-VCSELs, we evaluate the quality of generated random sequences using statistical and cryptographic metrics. NIST SP800-22 is designed to evaluate the statistical randomness of binary sequences [41]. The NIST SP800-90B statistical tests are applied to measure entropy, which quantifies the unpredictability of a system. Hamming distance analysis evaluates the uniqueness of generated keys by comparing bitwise differences between sequences, ensuring robustness against predictability. Additionally, autocorrelation analysis detects any dependencies between consecutive bits, while statistical bias tests determine whether the probability of ‘0’s and ‘1’s is evenly distributed, which is crucial for cryptographic security.

## 3. Results

### 3.1. Generation and Optimization of Broadband Polarization Chaos

Spin-VCSELs offer a unique advantage over conventional VCSELs by enabling polarization control via spin-polarized carrier injection. One of the most significant degrees of freedom in QD spin-VCSELs is the pump ellipticity parameter *P*, which directly influences the spin polarization of the injected carriers. By adjusting P≠0, chaotic regions are significantly extended, both in the solitary QD spin-VCSEL [45] and in the optically injected system [39]. This makes spin-VCSELs highly tunable for chaos-based applications.

Beyond *P*, two additional parameters that critically impact the emergence of chaos in spin-VCSELs are the birefringence rate $\gamma_{p}$ and the spin-flip rate $\gamma_{j}$. The birefringence rate $\gamma_{p}$ determines the polarization coupling strength within the cavity, while the spin-flip rate $\gamma_{j}$ governs the relaxation dynamics between spin-up and spin-down carrier populations. Smaller values of $\gamma_{p}$ and $\gamma_{j}$ generally favor chaotic behavior by destabilizing polarization states [45]. However, achieving higher $\gamma_{p}$ values is also beneficial, as the oscillation frequency in spin-VCSELs is primarily dictated by $\gamma_{p}$ [30]. The interplay between high-frequency oscillations, which are influenced by birefringence-induced frequency splitting, and the onset of chaos plays a crucial role in pushing the chaotic bandwidth to higher values.

Additionally, QD physics introduce an extra degree of freedom that can be tuned through cavity and material engineering, the normalized gain coefficient *h* [46]. Li et al. demonstrated extended chaotic regions when *h* varied from 1.1995 to 2, highlighting the importance of this parameter for chaos enhancement [47].

Based on these insights, we select the following spin-flip model (SFM) parameters to model the QD spin-VCSEL under optical injection: $\beta_{sp}=0.0065$, $\gamma_{p}=30$ ns^−1^, $\gamma_{j}=10$ ns^−1^, $\gamma_{0}=400$ ns^−1^, $\gamma_{a}=0$ ns^−1^, h=2, $\gamma_{n}=1$ ns^−1^, k=250 ns^−1^, η=3 and $k_{inj}=200$ ns^−1^ [47].

**Figure 1:**
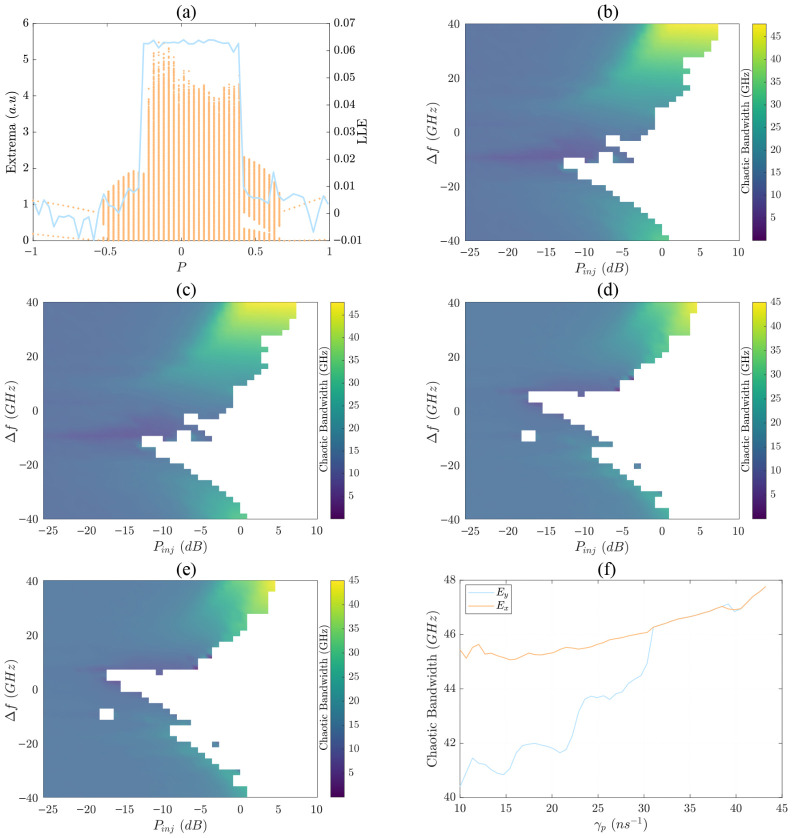
(**a**) Bifurcation diagram of the emitted intensity extrema as a function of pump ellipticity *P* with the LLE overlaid. (**b**–**e**) Chaotic bandwidth heatmaps of the emitted polarization components under different optical injection polarization conditions: (**b**) orthogonal injection ($E_{x}$), (**c**) parallel injection ($E_{y}$), (**d**–**e**) elliptical injection ($E_{y}$ and $E_{x}$). (**f**) Chaotic bandwidth as a function of birefringence rate $\gamma_{p}$ for the emitted polarization components $E_{x}$ and $E_{y}$. The white regions in the heatmaps correspond to non-chaotic regions, where LLE<0.04.

We present here how increasing the ellipticity of the circularly polarized pump (|P|=1) leads to extended chaotic regions, as observed in the bifurcation diagram of Figure 1a. The LLE follows a similar trend with qualitative chaos measurements (LLE≥0.04 [45]), which we use as the threshold for defining chaotic regions in the heatmaps of Figure 1b–e.

To examine the impact of different optical injection schemes, we vary the polarization angles $\theta_{p}$ and ϕ and investigate three scenarios: orthogonal injection (Figure 1b), parallel injection (Figure 1c), and elliptical injection (Figure 1d,e). For parallel injection $(\theta_{p}=0^{\circ }$ and $\delta =90^{\circ })$ and orthogonal $(\theta_{p}=90^{\circ }$ and $\delta =0^{\circ })$ injection, we present the chaotic bandwidth maps for the favorable emitted polarization component, $E_{y}$ or $E_{x}$, respectively. In both cases, we observe that high $E_{\mathrm{inj}}$ and large $\Delta_{f}$ lead to a maximum chaotic bandwidth of approximately 45 GHz.

An important observation is that elliptical injection $(\theta_{p}=45^{\circ }$ and $\delta =45^{\circ })$ extends this chaotic bandwidth to both polarization components. As seen in Figure 1d,e, elliptical injection enables both $E_{x}$ and $E_{y}$ to support a broadband chaotic bandwidth rather than confining it to a single polarization component as in parallel and orthogonal injection.

Additionally, we analyze the importance of the birefringence rate $\gamma_{p}$ by presenting an optimization analysis of chaotic bandwidth within the range where the system remains chaotic for the pair of $P_{inj}$ and $\Delta_{f}$ ($P_{inj}$ = 3 dB and $\Delta_{f}$ = 40 GHz). Figure 1f shows that for increasing $\gamma_{p}$, both components reach their maximum bandwidth at approximately 43 ns^−1^. Furthermore, we observe that as $\gamma_{p}$ increases, the chaotic bandwidths of both polarization components become more equalized, indicating that both $E_{x}$ and $E_{y}$ contribute equally to the total emission.

**Figure 2:**
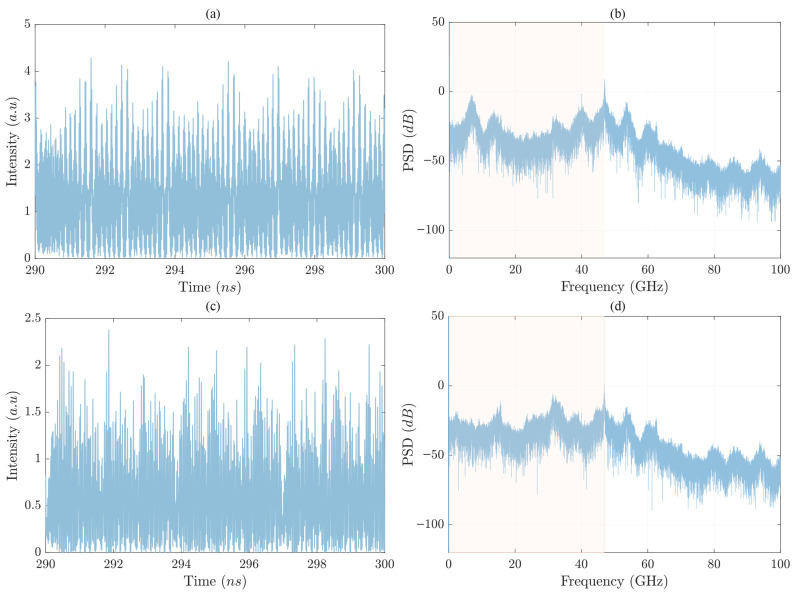
(**a**,**c**) Time series of the emitted polarization components $E_{x}$ and $E_{y}$ under elliptical optical injection for $\gamma_{p}=43$ ns^−1^. (**b**,**d**) Corresponding Power Spectral Density (PSD) plots in dB, illustrating the frequency distribution of the chaotic signal. The light yellow region in the spectrum represents the bandwidth containing 80% of the total spectral power.

To further analyze the chaotic emission properties, we present the time-domain waveforms and corresponding PSDs of the chaotic signals in Figure 2 for $\gamma_{p}=43\;{\mathrm{ns}}^{-1}$. The chaotic time traces confirm the broadband nature of the polarization-resolved emission, while the frequency-domain representation provides insight into the spectral distribution of the chaotic power. In the PSD plots, we highlight in yellow the spectral region that contains 80% of the total spectral power.

These results demonstrate that broadband polarization chaos can be achieved through appropriate optimization of injection parameters and birefringence control. Such broadband chaotic regimes may be exploited in optical sensing systems, where multi-GHz bandwidths can support centimeter-level ranging accuracy in correlation-based architectures. In parallel, they may also benefit secure communication platforms by enabling high-speed physical-layer encryption and polarization-multiplexed operation.

In the present work, however, we focus on the application of broadband polarization chaos for ultrafast RNG and photonic encryption. Notably, the parameter regimes yielding the maximum bandwidth do not necessarily correspond to the highest LLE values. Therefore, using the LLE as an initial filtering criterion for the NIST SP800-22 and SP800-90B evaluations presented in the next section, we operate the QD spin-VCSEL in a regime characterized by a slightly reduced effective bandwidth of approximately 40 GHz but significantly enhanced chaotic strength (higher LLE).

### 3.2. Entropy Evaluation of Broadband Polarization Chaos

**Figure 3:**
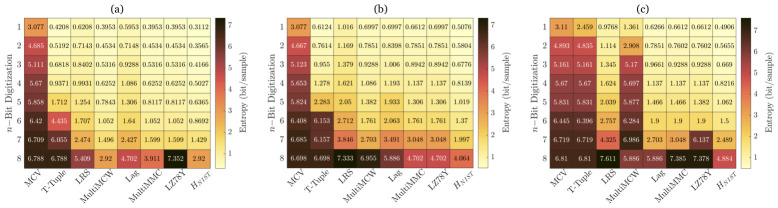
Results of the NIST SP800-90 tests applied to the extracted random bit sequences under three different polarized optical injection conditions. (**a**) Orthogonal polarization injection, (**b**) parallel polarization injection, and (**c**) elliptical polarization injection. The heatmaps display the entropy per bit sample across different statistical tests, with higher values indicating better randomness.

We assess the reliability of our entropy estimation using the NIST SP800-90B framework, which serves as the certified statistical tests for evaluating entropy sources in physical RNG [40]. This test suite applies ten different entropy estimation methods to quantify the unpredictability of the generated sequences, including the most common value (MCV), collision, Markov, compression, t-Tuple, longest repeated substring (LRS), multi-most common in window prediction (MultiMCW), lag prediction, MultiMMC prediction, and LZ78Y prediction. Since the collision, Markov, and compression estimators are designed specifically for binary data, they are excluded from our analysis, leaving the remaining seven estimators for evaluation in Figure 3. To ensure a conservative assessment of randomness, the minimum entropy value among these estimators is selected as the final estimate, denoted as $H_{NIST}$.

For this analysis, n-bit signals are extracted from the 8-bit digitized data, focusing on the most significant bits (MSBs) to examine entropy variations. The entropy is evaluated using 1 million samples per test configuration. The theoretical maximum entropy for each n-bit selection is n bits per sample, with deviations from this ideal value indicating potential biases or dependencies within the generated sequence.

Figure 3 presents the seven applicable NIST SP800-90B entropy estimates across different optical injection schemes. As expected, entropy increases with n for all estimators, suggesting that higher digitizing resolution provides a more refined measure of randomness by capturing additional uncorrelated fluctuations in the data. The results of the minimum entropy demonstrate that elliptical and parallel injection configurations exhibit strong entropy performance, consistent with previously published findings [48,49]. In contrast, the orthogonal injection scheme yields lower entropy values, suggesting weaker randomness characteristics compared to the other configurations. This highlights the advantage of elliptical and parallel injection schemes for high-speed, high-entropy RNG.

### 3.3. Random Bit Extraction and Statistical Validation

The process of bit extraction from the chaotic polarization-resolved signals generated by the QD spin-VCSEL under optical injection was introduced in our previous work [39], where we introduced the schematic for random bit extraction. The workflow is as follows: after detection of the chaotic optical signals corresponding to the orthogonal and parallel polarization components by photodetectors (PDs), the signals are digitized by 8-bit Analog-to-Digital Converters (ADCs) operating at 80 GSamples/s. The three least significant bits (LSBs) of each channel are then selected to ensure a high degree of randomness while maintaining an optimal trade-off between entropy extraction and statistical balance. The 80 GSamples/s sampling rate ensures operation well above the Nyquist rate, preserving the full chaotic bandwidth. To further enhance the randomness, the two extracted bit streams from the orthogonal and parallel components are combined using an Exclusive-OR (XOR) gate, resulting in a single random bit sequence. This combined process of sampling at 80 GSamples/s and extracting three LSBs per channel leads to an overall RNG rate of 240 Gb/s.

**Figure 4:**
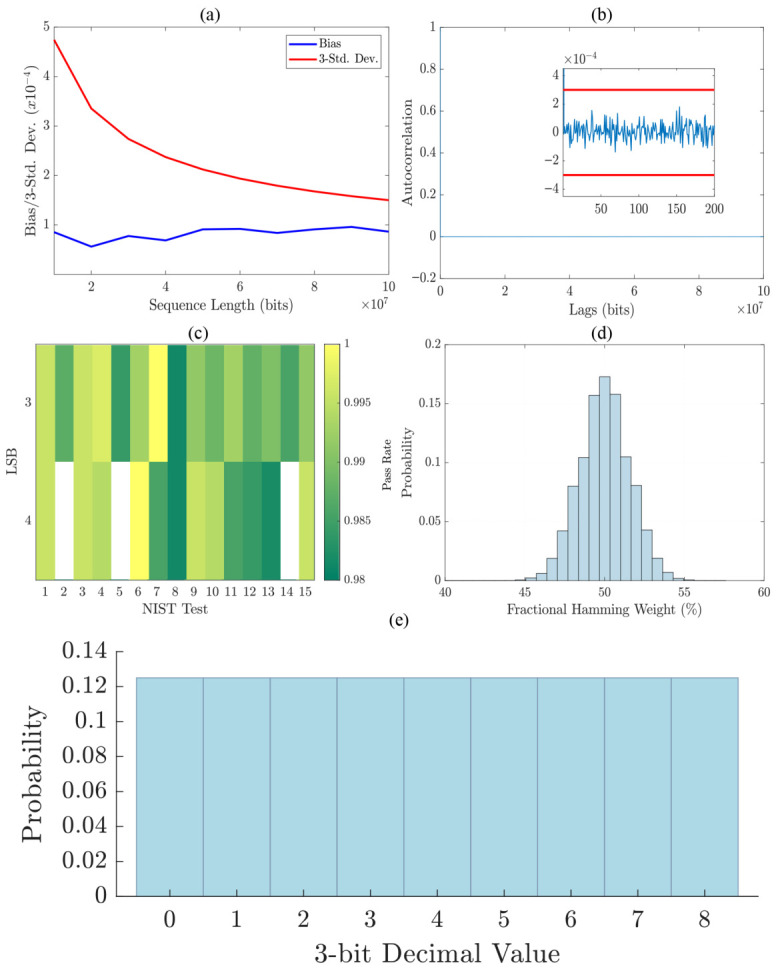
(**a**) Statistical bias and three-standard-deviation trends as a function of sequence length. (**b**) Autocorrelation function. The inset provides a zoomed-in view within the 3-standard-deviation bounds. (**c**) Pass rate for NIST SP800-22 tests as a function of LSB selection. The failed tests with a pass rate lower than 98% are indicated in white. (**d**) Hamming weight distribution for 1024-bit sequences. (**e**) Histogram of the 3-LSB decimal values.

To evaluate the statistical quality of the extracted random bits, we analyzed bias, autocorrelation, Hamming distance, statistical distributions, and pass rates for standard randomness tests, as presented in Figure 4.

Figure 4a illustrates the statistical bias and its deviation over increasing sequence lengths. The bias remains within acceptable limits, gradually decreasing as the sequence length increases, demonstrating the stability of the extracted random bits. Figure 4b presents the autocorrelation function, showing no significant correlation at any lag, ensuring the independence of the generated bits. The inset provides a zoomed-in view, confirming that all values remain within the three-standard-deviation bounds.

Figure 4d presents the pass rate for the NIST SP800-22 test suite as a function of the LSB for the elliptically polarized injection that yielded the highest entropy value in the previous subsection. Further details regarding this test suite are provided in the next subsection. The results demonstrate that three-LSB extraction maintains an optimal pass rate across all statistical tests. In contrast, four-LSB extraction results in three tests (indicated in white on the heatmap) failing to meet the 98% pass rate threshold.

To further validate randomness, Figure 4e displays the histogram of the extracted three-LSB decimal values, indicating a uniform distribution, which is crucial for avoiding statistical bias in RNG applications. Figure 4d depicts the fractional Hamming weight distribution for 1024-bit sequences, demonstrating a near-Gaussian distribution centered around 50%, confirming that the bit sequences exhibit high entropy and balance between ‘0’ s and ‘1’ s.

### 3.4. NIST SP800-22 Statistical Testing of Extracted Bits

This section presents the results of the NIST SP800-22 statistical test suite, a widely recognized benchmark for validating the statistical randomness of bitstreams. The test suite consists of 15 statistical tests [41] designed to detect potential non-random patterns. Each test was conducted using 1000 samples of a 1 Mbit sequence, applying a statistical significance level of α = 0.01. To meet the passing criteria, each *p*-value must exceed 0.0001, and the proportion must fall within the expected interval 0.99±0.0094. For multi-output tests, the worst-case results are reported to ensure a rigorous evaluation [14].

As shown in Table 1, all random bitstreams generated under elliptical injection passed the full suite of NIST SP800-22 statistical tests. Combined with strong performance in the NIST SP800-90B entropy evaluation, these results support the use of optically injected QD spin-VCSELs as certified physical entropy sources for secure communication and cryptographic key generation.

## 4. Conclusions

In this work, we presented a comprehensive characterization of the nonlinear dynamics of QD spin-VCSELs under optical injection and investigated their use as a physical entropy source for ultrafast random number generation. By exploiting polarization chaos dynamics, broadband chaotic bandwidths approaching 50 GHz per polarization component were achieved, corresponding to nearly 100 GHz of usable chaotic bandwidth in total. The observed high-speed chaotic behavior was systematically analyzed using bifurcation diagrams, Lyapunov exponent analysis, and chaotic bandwidth maps. The extracted random bit sequences were evaluated using commonly employed metrics in the relevant literature, while cryptographic suitability was assessed through the NIST SP800-22 statistical test suite. These results confirm that the proposed QD spin-VCSEL system can generate random bits at rates of up to 240 Gb/s. Furthermore, with optimized post-processing techniques or by employing dual-state QD spin-VCSELs capable of generating four independent chaotic signals, even higher random bit generation rates appear feasible. Overall, these findings establish QD spin-VCSELs as a strong candidate for future high-speed secure communication and cryptographic systems.

## Figures and Tables

**Table 1:** NIST SP800-22 test results.

Statistical Test	Orthogonal Injection		Result	Parallel Injection		Result	Elliptical Injection		Result
	*p*-Value	Proportion		*p*-Value	Proportion		*p*-Value	Proportion	
Frequency	0.6226	0.991	Success	0.0800	0.994	Success	0.6500	0.990	Success
Block frequency	0	0.989	Failure	0.2207	0.989	Success	0.1280	0.987	Success
Runs	0.3436	0.990	Success	0.0431	0.992	Success	0.3100	0.988	Success
Longest run	0.0800	0.996	Success	0.1163	0.985	Success	0.0750	0.995	Success
Rank	0.5980	0.988	Success	0.0889	0.992	Success	0.6001	0.989	Success
FFT	0.0489	0.991	Success	0.9496	0.992	Success	0.0512	0.990	Success
Nonoverlapping template	0.6181	0.986	Success	0.6918	0.989	Success	0.6230	0.987	Success
Overlapping template	0.0034	0.980	Success	0.0028	0.981	Failure	0.0040	0.979	Success
Universal	0.6673	0.991	Success	0.2884	0.992	Success	0.6600	0.990	Success
Linear complexity	0.6785	0.983	Success	0.7943	0.989	Success	0.6750	0.984	Success
Serial	0	0.987	Failure	0.2050	0.991	Success	0.7800	0.986	Success
Approximate entropy	0.0521	0.983	Success	0.0265	0.979	Success	0.0540	0.982	Success
Cumulative sums	0.0446	0.988	Success	0.0410	0.985	Success	0.0450	0.987	Success
Random excursions	0.0233	0.983	Success	0.7759	0.985	Success	0.0225	0.982	Success
Random excursions variant	0.4128	0.988	Success	0.0153	0.984	Success	0.4150	0.987	Success

## Data Availability

Data will be made available on request.

## Abbreviations

The following abbreviations are used in this manuscript:

Eqs.	Equations
Eq.	Equation
FFT	Fast Fourier Transform
LLE	Largest Lyapunov Exponent
LCP	Left Circular Polarized
NIST	National Institute of Standards and Technology
PSD	Power Spectral Density
QD	Quantum-Dot
ROF	Relaxation Oscillation Frequency
RCP	Right Circular Polarized
SFM	Spin Flip Model
Spin-VCSELs	Spin Vertical-Cavity Surface-Emitting Lasers
RBG	Random Bit Generator
RNG	Random Number Generation
VCSELs	Vertical-Cavity Surface-Emitting Lasers
